# Changes of Reaction Time and Blood Lactate Concentration of Elite Volleyball Players During a Game

**DOI:** 10.2478/v10078-011-0024-y

**Published:** 2011-07-04

**Authors:** Dariusz Mroczek, Adam Kawczyński, Jan Chmura

**Affiliations:** 1 Department of Athletes Motor Skills, Sport Institute, University School of Physical Education, Wrocław, Poland

**Keywords:** psychomotor fatigue threshold, reaction time, volleyball

## Abstract

The purpose of this study was to investigate changes in reaction time of elite volleyball players during a game. Fourteen volleyball players participated in the study. Reaction time was measured using the Optojump system. In addition, blood lactate concentration was assessed to monitor physiological load during the game. All measurements were performed during a pre-game test and during sets 1, 2, 3 and 4. Reaction time during set 1 decreased significantly by 13,3 % compared with pre-game values, from 600 ms during the pre-game test to 520 ms during set 1 (p<0,05). Blood lactate concentration increased significantly during set 1, 2, 3 and 4 compared with pre-game conditions (p<0,05). Reaction time stays in the first phase of its changes pattern and elite volleyball players do not reach psychomotor fatigue threshold throughout the game.

## Introduction

The ability to maintain psychomotor performance during a game is one of the most important aspect in sport competition (Chmura et al., 2010). Psychomotor performance’s definition includes the following components: reaction time (Chmura et al., 2010), choice reaction time (Chmura et al., 1994), movement time (McMorris et al., 2005), visual search (Tomporowski, 2003) and also motor skills e.g. running speed (Chmura et al., 2010).

In our experiment, we focused on reaction time (RT) changes and blood lactate concentration (LA) during the game. We decided to asses those parameters because fatigue, as a psycho-physiological state, exists at two levels: 1) peripheral - metabolic changes in working muscles limiting performance (Fitts 1994); 2) central – changes in the central nervous system which affect motor and perceptual processing and are extremely important in high level ball game participation (McMorris et al., 1997; Royal et al., 2006).

It must be underlined that there is a lack of research performed during training and sport competition. Most studies focused on the effect of exercise on components of psychomotor performance in laboratory settings and usually were performed in post-exercise conditions (Coles et al., 2008; Hillman et al., 2003; Kamijo et al., 2007; Kjaer, 1989; Themanson et al., 2006; Tomporowski, 2003). Those experiments aimed at simple cognitive tasks e.g. choice reaction time, visual search, which are basic components of complex decisions and actions performed by players during the game. Simple tasks activate different brain areas then complex decisions taken during competition (Tomporowski, 2003). Only competition and training require participants to perform activities of moderate to high intensities, with speed and accuracy (Chmura et al., 2010) and substantially affect athletes’ motor abilities (Aune et al., 2008).

Moreover, our previous studies performed in laboratory set-up showed that psychomotor performance assessed as reaction time during incremental exercise on treadmill (Chmura et al. 2010) and bicycle ergometer (Chmura et al., 1994) showed a biphasic pattern: that is the gradual shortening until power output of approximately 60–80% of maximal workload was attained and then a rapid increase in reaction time in the final stages of the exercise. We defined workload associated with the shortest RT as a “psychomotor fatigue threshold”.

This short literature analysis shows that investigation of RT of elite volleyball players during the game can provide new information in this area of research. Therefore, we hypothesized that it will be a difference between RT changes in laboratory set-up and during the volleyball game. The aim of this study was to evaluate this hypothesis.

## Material and methods

### Subjects

A total of 14 elite volleyball players participated in the study. The subjects included members of the Polish National Junior Team. The average age was 18 ± 1 years (mean ± SD), the average body height was 196 ± 7,39 cm, the average body weight equaled to 84,07 ± 7,77 kg. Informed consent was obtained from each subject. Experiment was performed during a preparation camp before the 2009 FIVB Boys Youth Volleyball World Championship, Jesolo-Bassano del Grappa, Italy. The study was approved by the Ethical Committee at the University School of Physical Education in Wroclaw.

### Blood lactate measurement

Blood samples were taken from the ear lobe for determination of blood lactate concentration using portable lactate test analyzer (Lactate Scout, SensLab GmbH, Leipzig, Germany). Blood lactate concentration was measured during pre-game conditions and during sets 1, 2, 3 and 4. Blood lactate concentration measurements were taken before RT measurements.

### Reaction time measurement

**Figure 1:**
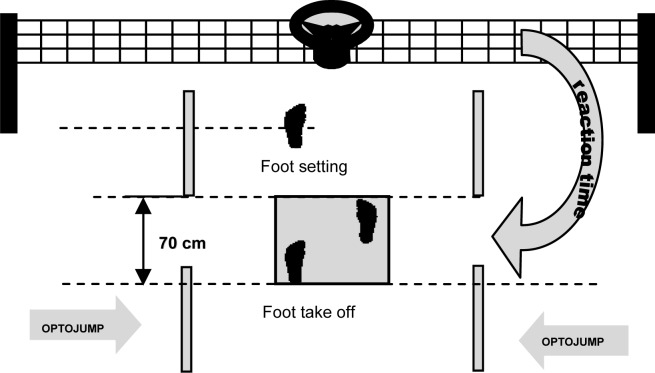
Reaction time measurement

The research employed the Optojump system (Microgate, Bolzano, Italy) to assess reaction time (RT) (Bosquet et al., 2009; Di Cagno et al., 2009). RT was measured during pre-game conditions and during sets 1, 2, 3 and 4. Before a pre-match warm up, all players were instructed in detail about the experiment procedure and location of measurement devices (Fig.1). The pre-game test was performed before the warm-up. Players performed a standardized volleyball warm-up: time 20 min, intensity at heart rate between 140 and 170 beat/min. After familiarization, during the pre-game test the resting value of RT and LA were assessed for all players participating in the experiment. With consideration to the extremely precise and dynamic nature of the whole trial, appropriate verbal motivation was also important for correct performance. Following verbal motivation: ”go as fast as possible” was the same for all participants. During the pre-game test and during the game, that is, during each of the four sets played, all players were subjected to an identical test procedure during which the player took his place in the starting square located on the base line of the court (Fig.1). The trial began inside a 70 cm square, where the subject assumed a staggered stance, without a defined lead foot.

The setting allowed for placement of backward leg so that the heel of the foot was always in the measurement area of the first couple of the Optojump measurement device (10 cm measuring from the tuber calcanei). Such a footing facilitated precise measurement of RT. The tested player stood facing the net, on which a light source was located at a height of 243 cm (stimulus), directly in front of the subject, who reacted to the signal by running as quickly as possible towards the light. A light stimulus was generated randomly (between 1 and 3 seconds) by the measuring system and each stimulus lasted 1 second.

Electronic time measurement commenced with the light source illuminating. At exactly this moment, the light source supply activated a relay responsible for the sending of an impulse to the software (Fig. 1). This impulse was registered by the Optojump software. Electronic time measurement ended with the removal of the heel of foot from the area located in the first module couple of the Optojump system. RT was measured during pre-game test and during sets 1, 2, 3 and 4.

### Statistics

Reaction time and blood lactate concentration were analyzed using repeated-measures analysis of variance (RMANOVA) between sets (pre-game test and set 1, 2, 3, 4). The significant results were analyzed further using simple contrast (compared with pre-game test and set 1). The normality of data distribution was checked by Shapiro-Wilk W test. The significance level *p* was set at 0.05. The data are presented as means with standard errors (SEM).

## Results

### Reaction time

**Figure 2:**
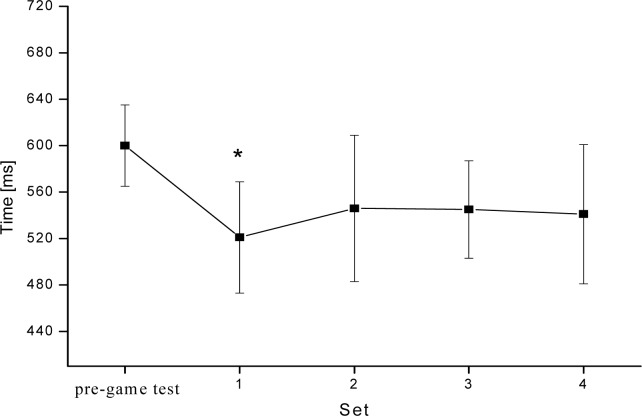
Time course changes of reaction time (mean ± SEM) for each set of the game. * Significant decrease compared with the pre-game test.

The RMANOVA revealed that volleyball game had an effect on RT. During set 1 RT decreased significantly by 13.3 % compared with the pre-game test (from 600±40 to 520±50 ms, F_(4,52)_ = 0.57, p<0.05). RT also decreased by 8.3% during set 2 and 3 (to 550±60 and 550±40 ms respectively) and by 10% during set 4 (to 540±60 ms). Those decreases were not statistically significant compared with the pre-game test (p>0.05). Differences between RT during set 1 and during sets 2, 3, 4 were not statistically significant (p>0.05) (Fig.2.; Tab.1).

### Blood lactate concentration

**Figure 3:**
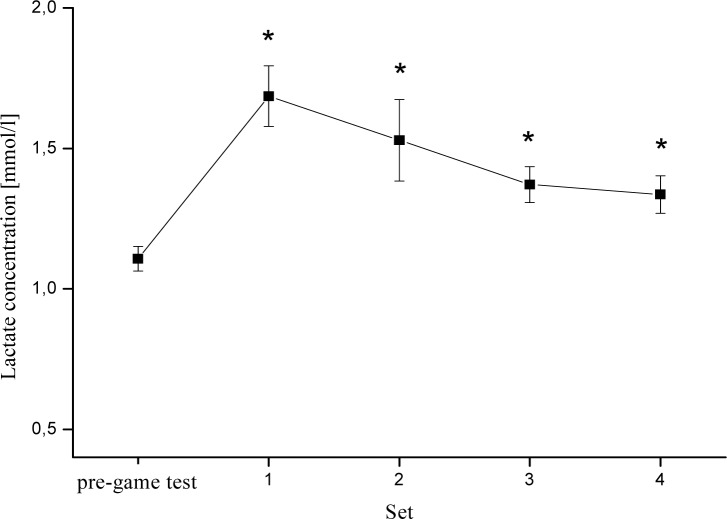
Time course changes of blood lactate concentration (mean ± SEM) for each set of the game. * Significant increase compared with pre-game test.

As expected, the lactate concentration in blood (LA) increased significantly during set 1, 2, 3 and 4 compared with pre-game test (p<0.05). LA increased from 1.1±0.04 to 1.7±0.11; 1.5±0.15; 1.4±0.06 and 1.3±0.07 during set 1, 2, 3 and 4 respectively (Fig.3; Tab.1).

## Discussion

The present study performed during the game showed reaction time and blood lactate concentration changes. Data obtained clearly showed that reaction time shortened during the game, which confirms previous results showing that exercise affects reaction time (Chmura et al., 2010; Chmura et al., 1994). As expected, blood lactate concentration increased significantly.

The new finding of the present study is that the RT of elite volleyball players shortens during the game and stays in the first phase of RT changes. This finding confirmed our hypothesis that there is a difference between RT changes in laboratory set-up and during the volleyball game. A biphasic pattern of RT changes was previously found during incremental exercise on treadmill (Chmura et al., 2010) and bicycle ergometer (Chmura et al., 1994). During the first phase RT shortens and elongates during the second phase after reaching the psychomotor fatigue threshold. Moreover, there is a high positive correlation between onset of blood lactate accumulation (OBLA) and psychomotor fatigue threshold (Chmura et al., 2010). OBLA is defined as the exercise load during which lactate concentration in blood attains 4 mmol l^−1^ (Heck et al., 1985). In our study, the highest LA level was about 1.7 mmol l^−1^ (maximal individual blood lactate concentration was 3.2 mmol l^−1^) and indicates that during the game players did not reach OBLA and psychomotor fatigue threshold. Low values of LA during the volleyball game in our experiment is the effect of game characteristic. Players change their position a during game and leave field to change their teammates. It results in recovery and rather low level of physiological parameters in spite of high level competition. In general, in a volleyball game low concentrations of lactate (2.54 +/− 1.21 mmol/l) during and after matches and increase of free fatty acids indicate that energy during the short exercise periods is mainly supplied by a breakdown of creatine phosphate, while aerobic pathways restore the energy sources during rest periods (Kunstlinger et al., 1987).

McMorris et al. (2005) implemented testing of RT during whole body movement, which is partly similar to players activity during volleyball game. Subjects had to perform a run through obstacles and choose one of possible ways, as a reaction to different light signals. This psychomotor test was performed in three conditions: 1) after sitting in a chair to reach resting heart rate; 2) after cycling on a cycloergometer at 70% maximal power output (W_max_); 3) after cycling at 100% W_max_. Authors showed that after exercise at 70% W_max_, where mean lactate concentration in blood was about 3 mmol/l, RT was significantly shorter then after two other conditions. After cycling at 100% W_max,_, where mean lactate concentration in blood was 8 mmol/l, RT was significantly longer then after sitting in a chair. The above results presented that we can also recognize a biphasic pattern of RT changes. RT shortens after exercise at lactate concentration level below OBLA and elongates after exercise at lactate concentration above OBLA.

Shortened RT during the game in our study is in line with previous studies evaluating the influence of exercise on RT (Chmura et al., 2010; Chmura et al., 1994; McMorris et al., 2005), but it must be underlined that the physiological load during the game in our study had a different level. Moreover, those studies were carried out in laboratory settings not during real competition. Chmura et al. (2010) used a protocol in which subjects performed a treadmill test at 0 grade with running speed increasing every 3 min by 2 km/h, starting from 6 km/h until exhaustion. During another study of Chmura et al. (1994), subjects performed an incremental, multistage bicycle ergometer exercise test with work load increasing at a rate of 50 W, until volitional exhaustion. The exercise stages lasted 3 minutes and were separated by 1 min rest periods. In the study of McMorris et al. (2005), subjects had to perform a run through a slalom-type obstacle course. Mean run time to pass obstacle was about 1,5 seconds. To summarize this short literature analysis, the following facts should be underlined: 1) reaction time shortens during exercises with different load levels and characteristics; 2) exercise performed in the laboratory and during the game may have similar influence on reaction time; 3) the same pattern of reaction-time-changes in different conditions may suggest not only peripheral but also central origin of the pattern i.e. increased arousal and/or motivation.

The facts presented above are supported by recent studies which showed that exercise itself has an influence on central nervous parameters: 1) exercise increases the speed of response by energizing motor outputs indicated by EMG (Audiffren et al., 2008); 2) intensity of exercise has a significant linear effect on information processing speed (Chang et al., 2009).

When we want to explain changes of RT during a volleyball game, we have to remember about the mental state of the players. It is well-known that competition increases arousal (Rhea et al., 2003; Sibley et al., 2004). The relationship between the level of arousal and psychomotor skill is well documented in literature. This relationship is described as inverted “U”, which means that RT shortens with increase of central nervous system arousal. After passing this optimal level RT elongates (Arent et al., 2003; Duffy, 1957). Basing on findings presented above, we can assume that during the game shortening of RT can be an effect of increased arousal of the central nervous system (CNS) as the results of competition.

The limitation of this study is the lack of a control group consisting of volleyball players of lower sports level. Moreover, the variability of the measured psychomotor performance components should be assessed because the number of trials in the present study was limited by game requirements.

Practical application of our study are as follows: 1) RT decreased by 0,08 seconds, which allows players to cover about 30 cm distance during the game; 2) Elite volleyball players do not pass the psychomotor fatigue threshold during the game. This can be crucial in players’ efficiency and game success.

## Figures and Tables

**Table 1:** Reaction time and blood lactate concentration during a pre-game test and sets 1-4. Values are means ± SEM. Asterisks denote significant difference between values obtained in consecutive sets (1–4) as compared with pre-game test.

**Variable**	**Pre-game test**	**Set 1**	**Set 2**	**Set 3**	**Set 4**
Reaction time [ms]	600 ± 40	520 ± 50^*^	550 ± 60	550 ± 40	540 ± 60
Lactate conc. [mmol/l]	1.1 ± 0.04	1.7 ± 0.11^*^	1.5 ± 0.15^*^	1.4 ± 0.06^*^	1.3 ± 0.07^*^
